# Brighton Charities

**Published:** 1889-02-16

**Authors:** 


### A CHRISTMAS HOLIDAY.

*(Concluded from p. lxxi.)*

### IV.—BRIGHTON CHARITIES.

CHRISTMAS DAY and Boxing Day we spent quietly with our friends, and then came the fatal Thursday on which we had to return to work. But we were not due at the Home till nine p.m., so we determined to spend the day in looking at some of the homes and hospitals. Brighton just swarms with convalescent homes, and as we were on the cliff we visited Black Rock Home first. It is a melancholy-looking building from the outside; weather-worn and bare, and evidently well buffetted by stormy winds. But inside all is bright and cheery. To begin with, the lady superintendent gives you a smiling welcome, and what a difference that makes! I always drop something in the box of a charity that greets me kindly, even though it is only a sixpence; for I feel that courtesy should be repaid, and that is all I can do to show how I reciprocate the kindly feelings of those who are in power. The Black Rock Home consists of two houses, one for men and one for women. Large dormitories have been lately built on at the back, to give extra accommodation. The sitting-rooms are large, light, and supplied with easy chairs; the windows look towards the sea. Everything was very plain, but very clean, and we quite enjoyed our tour of inspection. When we got outside the doors again the old feeling that it was a bleak, dreary spot struck us; it is almost the last house at the east end of Brighton; and away to our left rose the treeless downs.

"Winter is a bad time to look at convalescent homes," said Nurse Sadler, "of course they don't look nice this weather. Let us go over the Sussex County Hospital."

"Well, yes," I said, "I should like that, I remember when I was in Brighton last summer I saw two nurses walking on the roof, and I knew at once it must be a nice hospital."

So we went along to the Sussex County, and were pioneered by the porter to the matron's room, and just as we got there a bevy of laughing nurses came trooping out, who had evidently been having a gossip with their matron. Once more we got a pleasant welcome, and Miss Scott kindly took us round all the wards. The decorations were very pretty, and in every ward was a circular flower-stand of several tiers. Just fancy what a lucky hospital to have plants enough to deck a stand! Most of us are content if we can get a palm for our window-sill, or central table. The beds were very high, so that the patients as they lay could look out at the sea. The hospital treats yearly over 1,000 in-patients and 8,000 out, so that it is a good school for nursing. There are two house-surgeons and one house-physician. There is a Samaritan fund and private nursing institute connected with the hospital. We certainly enjoyed our wanderings through the rambling wards: patients and nurses looked cheerful, and there was a homely air about the curtained beds and colour-washed walls. Tiles may be very healthy and pretty, but they are cold and cheerless-looking, and spoil the appearance of many modern hospitals. The only thing we missed at the Sussex County were the dainty little surgical tables now to be found in most accident wards.

When at last we bade good-bye to Miss Scott, we paid a hasty visit to St. John's Convalescent Home for Children, a new red building which stands on the downs close above the Sussex County. We found it a charming Home, presided over by a true Mother, and swarming with invalid children, who all turned affectionate eyes towards our black-robed guide. There was a large covered playground, fitted with swings, etc. We had not long to stay; the day was passing, our holiday nearly over. Well! it had been a most enjoyable holiday, in spite of our failure to complete our projected walk.

